# Impact of the COVID-19 pandemic on incidence of tics in children and young people: a population-based cohort study

**DOI:** 10.1016/j.eclinm.2023.101857

**Published:** 2023-02-16

**Authors:** Ruth H. Jack, Rebecca M. Joseph, Carol A.C. Coupland, Charlotte L. Hall, Chris Hollis

**Affiliations:** a Centre for Academic Primary Care, Lifespan and Population Health, School of Medicine, University of Nottingham, Nottingham, UK; b National Institute for Health Research (NIHR) Nottingham Biomedical Research Centre, Nottingham University Hospitals NHS Trust, Nottingham, UK; c Mental Health and Clinical Neurosciences, School of Medicine, University of Nottingham, Nottingham, UK; d NIHR MindTech MedTech Co-operative, Institute of Mental Health, School of Medicine, University of Nottingham, Innovation Park, Triumph Road, Nottingham, UK

**Keywords:** Tourette syndrome, Tics, COVID-19

## Abstract

**Background:**

Since the onset of the coronavirus (COVID-19) pandemic, clinicians have reported an increase in presentations of sudden and new onset tics particularly affecting teenage girls. This population-based study aimed to describe and compare the incidence of tics in children and young people in primary care before and during the COVID-19 pandemic in England.

**Methods:**

We used information from the UK Clinical Practice Research Datalink (CPRD) Aurum dataset and included males and females aged 4–11 years and 12–18 years between Jan 1, 2015, and Dec 31, 2021. We grouped the pre-pandemic period (2015–2019) and presented the pandemic years (2020, 2021) separately. We described the characteristics of children and young people with a first record of a motor or vocal tic in each time period. Incidence rates of tics by age-sex groups in 2015–2019, 2020, and 2021 were calculated. Negative binomial regression models were used to calculate incidence rate ratios.

**Findings:**

We included 3,867,709 males and females aged 4–18 years. Over 14,734,062 person-years of follow-up, 11,245 people had a first tic record during the whole study period. The characteristics of people with tics differed over time, with the proportion of females aged 12–18 years and the proportion with mental health conditions including anxiety increasing during the pandemic. Tic incidence rates per 10,000 person-years were highest for 4–11-year-old males in all three time periods (13.4 [95% confidence interval 13.0–13.8] in 2015–2019; 13.2 [12.3–14.1] in 2020; 15.1 [14.1–16.1] in 2021) but increased markedly during the pandemic in 12–18-year-old females, from 2.5 (2.3–2.7) in 2015–2019, to 10.3 (9.5–11.3) in 2020 and 13.1 (12.1–14.1) in 2021. There were smaller increases in incidence rates in 12–18-year-old males (4.6 [4.4–4.9] in 2015–2019; 4.7 [4.1–5.3] in 2020; 6.2 [5.5–6.9] in 2021) and 4–11-year-old females (4.9 [4.7–5.2] in 2015–2019; 5.7 [5.1–6.4] in 2020; 7.6 [6.9–8.3] in 2021). Incidence rate ratios comparing 2020 and 2021 with 2015–2019 were highest in the 12–18-year-old female subgroup (4.2 [3.6–4.8] in 2020; 5.3 [4.7–6.0] in 2021).

**Interpretation:**

The incidence of tics in children and young people increased across all age and sex groups during the COVID-19 pandemic, with a differentially large effect in teenage girls (a greater than four-fold increase). Furthermore, in those with tic symptoms, proportions with mental health disorders including anxiety increased during the pandemic. Further research is required on the social and contextual factors underpinning this rise in onset of tics in teenage girls.

**Funding:**

10.13039/501100000272National Institute for Health Research Nottingham Biomedical Research Centre.

**Research in context**

**Evidence before this study**

Tics are sudden involuntary movements and sounds which typically occur more commonly in boys, with a peak incidence between age 5 and 7 years. During the coronavirus (COVID-19) pandemic, clinicians have described in numerous case reports a phenomenon of sudden onset tics arising predominantly in teenage girls. These atypical presentations have been described as functional tics with a presumed association with pandemic related social stressors. One longitudinal study of the ORBIT trial participants, who all had pre-existing tic disorder diagnoses (chronic tic disorder including Tourette syndrome), found no increase in tic intensity during the pandemic. A search of PubMed using the terms “COVID-19” and (“tics” or “Tourette”) found no population-based studies examining the incidence of tics during the COVID-19 pandemic.

**Added value of this study**

Our study in a large population-based cohort of people aged 4–18 years found that the incidence of tics increased across all age (4–11, 12–18 years) and sex groups during the pandemic. However, there was a differentially large effect in teenage girls with a greater than four-fold increase in the incidence of tics during the pandemic. In children and young people with new onset tic symptoms, rates of anxiety, depression, self-harm and eating disorders all increased during the pandemic.

**Implications of all the available evidence**

This large population-based cohort study confirms the signal from clinical case reports of a marked rise in new onset tic presentations in teenage girls during the COVID-19 pandemic. The characteristics of these tic presentations suggest that this rise may be driven by the emotional and social impact of the pandemic on teenage girls and that functional tics should be considered as part of the clinical differential diagnosis.

## Introduction

Since the onset of the COVID-19 pandemic, clinicians a have noticed a marked increase in presentations of severe sudden and new onset tics and ‘tic-like’ attacks particularly affecting teenage girls.^1^^,^^2^ This phenomenon appears to have been recognised globally with case reports from the UK, Europe and the US^2^, ^3^, ^4^, ^5^, ^6^, ^7^ and widespread media coverage of this phenomenon, often emphasising the role of social media.^8^, ^9^, ^10^ Tics are defined as sudden, involuntary motor movements and vocal utterances that affect around 1% of children and young people with tics having a peak incidence between the ages of 5–7 years.^11^ Tic disorders (including Tourette syndrome) are typically more common in boys. The recent descriptions of sudden first onset tics in teenage girls differs from the typical age/sex onset pattern and characteristic of tics and tic disorders. In case reports and cohort studies, these teenage onset acute tic presentations are often considered to be functional in nature (described as ‘functional tics’ or functional neurological disorder (FND)),^12^ typically do not appear to respond to standard treatments for tic disorders and Tourette syndrome, and are thought to be related to increased levels of stress and anxiety experienced during the pandemic.^2^, ^3^, ^4^

The adverse impact of the COVID-19 pandemic on child mental health has become increasingly evident. In March 2020, the United Kingdom (UK) government enforced lockdown restrictions, including limiting social contact and home confinement for all but essential activities. Notwithstanding periodic relaxation of rules, restrictions remained in place until April 2021. Additionally, in the first three months of the pandemic (March–May 2020), school closures were mandated, affecting over 96% of school and college students in England.^13^ These social restrictions sparked major concerns about the impact on vulnerable children and young people's mental health as young people turned increasingly online for education, social contact and support.^14^ The follow-up of England's Mental Health of Children and Young People survey provided a rare resource on the impact of the pandemic, with pre-pandemic measures in a sample of 3570 children and young people.^15^^,^^16^ The study showed an increase in probable mental health problems reported in 5–16 year olds, with the incidence rising from 10.8% in 2017 to 16.0% in July 2020. Although the survey didn't report on tic symptoms, the reported increase in mental health problems and pandemic-related stressors could potentially act as a trigger in vulnerable children and young people either to exacerbate existing tic disorders or develop new onset tics, including functional tics.

To date there has been very little evidence available from large population datasets to confirm and quantify the signal from anecdotal clinical reports of increasing presentations of tics in teenage girls during the COVID-19 pandemic. A survey of parents of young people in Italy reported that 67% felt their children's tics had worsened since the pandemic.^5^ A large database review conducted in the US by the Centres for Disease Control and Prevention compared visits to the emergency department pre- and during the pandemic and revealed an increase in tics during 2020, 2021 and 2022 compared to pre-pandemic referrals, particularly in adolescent girls.^7^ In the UK, a chart review of 34 patients presenting with sudden onset tic disorders during the pandemic, found that 94% were female.^17^ The largest UK tic study utilising pre- and during pandemic tic scores followed 112 (78% male) children with tics who participated in the active control group of a randomised controlled trial (“ORBIT Trial”).^18^^,^^19^ The analysis revealed no difference in tic severity during the pandemic,^20^ although the sample was limited to children who were diagnosed with a tic disorder pre-pandemic. Taken together, these findings indicate a mixed, complex picture in which some groups of children and young people may be differentially at greater risk than others of developing tics during the COVID-19 pandemic.

To understand the impact of the COVID-19 pandemic on tic incidence, and potential differential effects of age and sex, there is a need to explore the recording of new tic symptoms in a population-based cohort both before and during the pandemic. To this end, this study aimed to describe and compare the incidence of tic symptoms in children and young people by age and sex groups in primary care records before and during the coronavirus (COVID-19) pandemic, and to describe associated mental health characteristics. We hypothesised that there was a differential increase in the incidence of tics in teenage girls associated with a rise in emotional symptoms during the pandemic.

## Methods

### Study design and participants

This was a cohort study set in England between Jan 1, 2015 and Dec 31, 2021. In England, access to most non-emergency healthcare via the National Health Service (NHS) is centralised through general practitioners (GPs) who are the first point of contact in the healthcare system. If necessary, patients are then referred to secondary care, and additional information from secondary care is sent back to GPs. The study used data provided by the Clinical Practice Research Datalink (CPRD),^21^ a database of anonymised primary care electronic health records (EHR) provided by participating general practices. The primary care data contain patient-level information including demographic characteristics and recorded symptoms and diagnoses, coded using a mixture of Read codes, proprietary EMIS® codes, and SNOMED CT codes. Symptom and diagnosis codes are recorded in the EHR by clinicians or other practice staff as part of routine clinical care, during or following consultations or other relevant contact with the healthcare system. For this study we used the CPRD Aurum dataset (March 2022 build), and linked Index of Multiple Deprivation (IMD) data (linkage based on patient and practice postal codes).^22^^,^^23^ Access to CPRD data is governed by their Research Data Governance process and this study was approved in January 2022 (reference 21_001650). Individual patient consent is not required for CPRD studies, as only anonymised information is shared with CPRD. However, people can request to opt out of sharing their data for research with CPRD.

The study included children and young people aged 4–18 years between Jan 1, 2015 and Dec 31, 2021. People joined the cohort on the latest date of turning 4 years old, first registration at the general practice plus one year, or Jan 1, 2015. Follow-up ended on the earliest date of turning 19 years old, end of registration with practice, last practice data collection date, or Dec 31, 2021. Person-years were calculated from cohort entry until either their first tic record or end of follow-up. People were excluded if they had a tic record before cohort entry, or if their sex was missing or not classified.

### Outcome

The study outcome was a coded record of tic symptoms (tic record) in the primary care data. To identify relevant records, we defined a list of codes for tics, based on an existing list of Read codes^24^ and adapted for use with the CPRD Aurum dataset (Supplementary Table S1). The code list was reviewed and agreed by a clinical expert (CH, a consultant child and adolescent psychiatrist) and is available online (see data sharing statement). The first coded record per person during the study window was identified.

### Covariates

For the denominator population we defined age group based on primary school (Years/Grade 1–6) and secondary school (Years/Grade 7–13) ages in the UK (4–11 years, 12–18 years), sex (male, female), deprivation score, and practice region. The patient-level IMD quintile was used to represent deprivation (2015 IMD for England, composite score),^22^ substituting the practice-level IMD quintile (2019 IMD for England, composite score)^23^ where the patient-level value was unavailable.

The remaining covariates were defined, with respect to the date of the first tic record, for the subset of the study population who had a tic record during the study period. These covariates were ethnicity (Asian/Asian British, Black/Black British, Mixed, Other, White, based on the England and Wales 2001 Census groups), and diagnoses of the following comorbidities: anxiety, autism spectrum disorder (ASD), attention deficit hyperactivity disorder (ADHD), depression, eating disorders, obsessive compulsive disorder, self-harm (intentional or unspecified), dissociative or somatoform disorder, and stress reaction or adjustment disorder. These comorbidities were defined as present if recorded at any time on or before the date of the tic record. Code lists to define these variables were adapted from existing publications.^24^, ^25^, ^26^, ^27^, ^28^, ^29^, ^30^, ^31^, ^32^, ^33^, ^34^, ^35^, ^36^ New code lists were developed for dissociative or somatoform disorder and stress reaction or adjustment disorder by searching code dictionaries for relevant terms based on definitions within the International Classification of Diseases (ICD-10) categories F43, F44, and F45. The final code lists were reviewed by CH, as above. All code lists are available online (see data sharing statement).

### Statistical analysis

We summarised the characteristics of children and young people with a tic record using descriptive statistics over three time periods: 2015–2019, 2020, and 2021, according to the year when the tic was first recorded. Incidence rates of tics per 10,000 person-years were calculated for these time periods, overall, by month, and in the four age-sex subgroups. The results were plotted as a time series, highlighting periods of government-mandated school closures in England. Negative binomial regression models were used to calculate incidence rate ratios accounting for overdispersion, including terms for time period, age group and sex. Interaction terms were included in the models to assess the interaction between time period and the four age-sex subgroups. A sensitivity analysis also adjusted for deprivation and region.

All data handling and analyses were conducted using Stata MP/17.0. A significance level of 0.05 was used throughout. Small cell counts in tables have been masked. The Stata do-files needed to replicate the data preparation and analysis are available to download (see data sharing statement).

### Role of the funding source

The funder of the study (National Institute for Health Research) had no role in study design, data collection, data analysis, data interpretation, or writing of the report.

## Results

Between Jan 1, 2015 and Dec 31, 2021, 3,869,667 people aged 4–18 years had eligible follow-up within CPRD. After excluding 1788 people who had a tic record before study entry and 170 people with missing or unclassified sex information, the final study population included 3,867,709 people aged 4–18 years. Over a total of 14,734,062 person-years of follow-up, 11,245 people had a first tic recorded during the study period (7113 during 2015–2019, 1861 in 2020 and 2271 in 2021). Overall, 7393 (65.7%) people with a tic record were male and the median age at first tic record was 9 years (interquartile range 7–12 years).

The characteristics of people according to the year of their first tic record are summarised in Table 1. Among people with an incident tic record, the proportion who were 12–18-year-old females increased from 7.5% of the total in 2015–2019, to 26.3% in 2020 and 27.7% in 2021. There were also increases in the proportion of people with a tic record who also had records of anxiety, depression, self-harm, and eating disorders. The proportion with anxiety recorded increased from 5.7% in 2015–2019 to 12.1% in 2020 and 13.0% in 2021 (Table 1). As shown in Supplementary Table S2, the increase in anxiety was seen in both male age groups and in 12–18-year-old females. The proportion of people with tics who also had ADHD recorded did not change significantly over time, except for in 12–18-year-old females, for whom the proportion with ADHD decreased from 6.2% in 2015–2019 to 2.9% in 2020 and 4.9% in 2021. Due to small numbers, it was not possible to break down all conditions by age and sex.

**Table 1:** Characteristics of children and young people aged 4–18 years according to the year of their first primary care tic record (2015–2019, 2020, 2021).

	Year of first tic record						P value
	2015–2019		2020		2021		
	N	%	N	%	N	%	
Number of people	7113		1861		2271		
Age-sex group							
Males 4–11 years	4067	57.2%	804	43.2%	899	39.6%	
Males 12–18 years	1078	15.2%	234	12.6%	311	13.7%	
Females 4–11 years	1435	20.2%	334	17.9%	433	19.1%	
Females 12–18 years	533	7.5%	489	26.3%	628	27.7%	
Ethnicity^a^							
Asian/Asian British	336	6.4%	75	5.3%	80	4.6%	
Black/Black British	122	2.3%	27	1.9%	39	2.3%	
Mixed	183	3.5%	55	3.9%	64	3.7%	
Other	73	1.4%	20	1.4%	33	1.9%	
White	4537	86.4%	1230	87.4%	1506	87.5%	
Missing ethnicity	574	29.2%	213	25.9%	263	24.8%	
Practice region							
North East	255	3.6%	66	3.5%	71	3.1%	
North West	1190	16.7%	343	18.4%	367	16.2%	
Yorkshire and the Humber	232	3.3%	73	3.9%	88	3.9%	
East Midlands	180	2.5%	37	2.0%	50	2.2%	
West Midlands	1021	14.4%	259	13.9%	326	14.4%	
East of England	406	5.7%	106	5.7%	116	5.1%	
London	976	13.7%	220	11.8%	323	14.2%	
South East	1803	25.3%	477	25.6%	572	25.2%	
South West	1050	14.8%	280	15.0%	358	15.8%	
Deprivation quintile (IMD)^a^							
1 (least deprived)	1752	25.1%	419	22.7%	491	21.8%	
2	1421	20.3%	396	21.5%	457	20.3%	
3	1294	18.5%	351	19.0%	451	20.1%	
4	1207	17.3%	345	18.7%	419	18.6%	
5 (most deprived)	1318	18.9%	334	18.1%	431	19.2%	
Missing deprivation	121	1.7%	16	0.9%	22	1.0%	
							
ADHD	680	9.6%	146	7.8%	195	8.6%	0.048
Anxiety (phobic or generalised)	407	5.7%	226	12.1%	296	13.0%	<0.001
Autism Spectrum Disorder	583	8.2%	158	8.5%	202	8.9%	0.570
Depression	56	0.8%	37	2.0%	59	2.6%	<0.001
Eating disorder	36	0.5%	17	0.9%	33	1.5%	<0.001
Obsessive compulsive disorder	5	0.1%	<5		<5		
Self-harm (intentional or unspecified)	69	1.0%	54	2.9%	77	3.4%	<0.001
Dissociative or somatoform disorder	22	0.3%	5	0.3%	9	0.4%	0.743
Stress reaction or adjustment disorder	20	0.3%	<5		13	0.6%	

N number, IMD index of multiple deprivation, ADHD attention deficit hyperactivity disorder. Small cell counts (<5) have been masked.

a Percentages exclude those with missing values.

### Incidence rates of tics

**Fig. 1:**
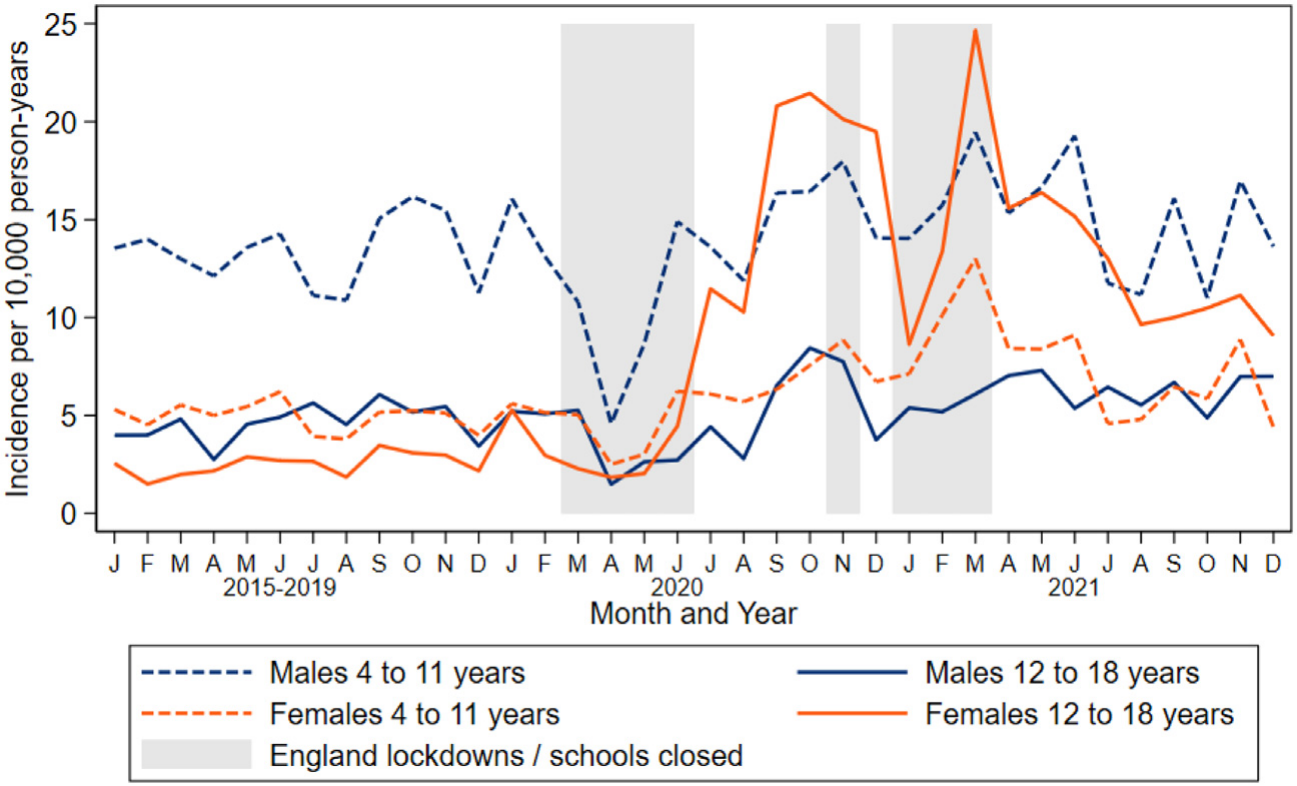
**Incidence rate of tics in England per 10,000 person-years by age and sex, in each month for 2015–2019, 2020, and 2021**.

Monthly incidence rates of tics over the study period are shown in Fig. 1, along with highlighted periods of lockdown and school closures in England. This shows initial decreases in rates at the start of the pandemic (March/April 2020) followed by increases in all four age-sex groups. The largest increase was in 12–18-year-old females, with the incidence rate increasing from 2.0 per 10,000 person-years in May 2020 to 4.5 per 10,000 person-years in June 2020, and then to 11.5 per 10,000 person-years in July 2020 when the first lockdown restrictions were eased. There was a further increase to around 20 per 10,000 person-years between September and December 2020. Incidence rates for this group reached a peak of 24.7 per 10,000 in March 2021 and then started to decline. They remained above pre-pandemic levels in December 2021.

The incidence rates of tics according to time period and age-sex group are summarised in Table 2 (incidence rates for individual calendar years are provided in Supplementary Table S3). Overall, the incidence rate was highest for 4–11-year-old males in all three time periods, and all four age-sex groups saw some increase by 2021. However, the largest change in incidence was in 12–18-year-old females, increasing from 2.5 per 10,000 person-years in 2015–2019 to 10.3 per 10,000 person-years in 2020, and 13.1 per 10,000 person-years in 2021.

**Table 2:** Number of people with a first tic record (events), person-years, incidence rate (IR) of tics per 10,000 person-years and 95% confidence interval (95% CI) by time period, age group, and sex.

	Year of first tic record								
	2015–2019			2020			2021		
	Events	Person-years	IR (95% CI)	Events	Person-years	IR (95% CI)	Events	Person-years	IR (95% CI)
Males 4–11 years	4067	3,043,344	13.36 (12.96–13.78)	804	609,478	13.19 (12.31–14.14)	899	595,815	15.09 (14.13–16.11)
Males 12–18 years	1078	2,335,329	4.62 (4.35–4.90)	234	500,118	4.68 (4.12–5.32)	311	504,897	6.16 (5.51–6.88)
Females 4–11 years	1435	2,908,778	4.93 (4.68–5.20)	334	583,440	5.72 (5.14–6.37)	433	570,444	7.59 (6.91–8.34)
Females 12–18 years	533	2,128,279	2.50 (2.30–2.73)	489	473,712	10.32 (9.45–11.28)	628	480,430	13.07 (12.09–14.14)

Results from the negative binomial regression model are shown in Table 3, with the full model in Supplementary Table S4. Incidence rate ratios (IRRs) for 2020 and 2021 compared with 2015–2019 were highest in the 12–18-year-old female subgroup (4.2, 95% CI 3.6–4.8, P < 0.001 in 2020; 5.3, 4.7–6.0, P < 0.001 in 2021). All other age-sex subgroups also had statistically significantly higher incidence rate ratios in 2021 compared with 2015–2019 (4–11-year-old females: 1.6, 1.4–1.8, P < 0.001; 4–11-year-old males: 1.1, 1.0–1.2, P = 0.004; 12–18-year-old males: 1.4, 1.2–1.6, P < 0.001). The 4–11-year-old females additionally had an increased IRR in 2020 (1.2, 1.0–1.3, p = 0.014) compared with 2015–2019, while both male age groups had no statistically significant change in rates in 2020. Additionally adjusting for deprivation and region did not materially affect the estimates for the age and sex groups (Supplementary Table S5).

**Table 3:** Unadjusted incidence rate ratios (IRR) of tics for time period by sex and age groups from negative binomial regression model.

	IRR	95% CI	P value
Males 4–11 years			
2015–2019	1.00		
2020	0.99	(0.91–1.09)	0.9137
2021	1.13	(1.04–1.24)	0.0043
Males 12–18 years			
2015–2019	1.00		
2020	1.03	(0.88–1.20)	0.7227
2021	1.35	(1.18–1.55)	<0.0001
Females 4–11 years			
2015–2019	1.00		
2020	1.18	(1.03–1.34)	0.0136
2021	1.55	(1.38–1.75)	<0.0001
Females 12–18 years			
2015–2019	1.00		
2020	4.16	(3.64–4.75)	<0.0001
2021	5.32	(4.68–6.04)	<0.0001

## Discussion

This large observational study found that during the COVID-19 pandemic the incidence of tic symptoms increased in children and young people across all age and sex groups. However, there was a markedly larger increase in teenage girls, with a greater than four-fold rise in the incidence of tics during the pandemic compared to pre-pandemic levels. The peak incidence in teenage girls occurred towards the end of the third national UK lockdown in March 2021. These population-based data support anecdotal clinical observations and case reports of a rise in the onset of tics in teenage girls during the pandemic. Furthermore, in those children and young people with tic symptoms, the proportions with anxiety, depression, self-harm, and eating disorders all increased during the pandemic, suggesting that the emotional and social impacts of the pandemic may be an important factor in this marked rise of tics in teenage girls.

The main strengths of this study were first, a very large sample (over 3.8 million children and young people) representative of the general population aged 4–18 years. Second, a study design that captured all coded records of incident tic symptoms in the primary care dataset during a pre-pandemic baseline period and during the pandemic. Third, the study included measures of mental health symptoms (e.g. anxiety, depression, and self-harm) and co-occurring conditions (e.g. ADHD, ASD, obsessive compulsive disorder and eating disorders) in the primary care record. Fourth, the size of the sample allowed incidence rate ratio comparisons to be made between age and sex subgroups of children and young people.

Our study has several limitations; first, there is uncertainty about the accuracy and completeness of tic symptoms recorded in primary care. New onset tics in children and young people are typically referred for assessment in secondary care. Furthermore, people may initially present to hospital emergency departments, as seen in the United States, where increased numbers of teenage girls were described presenting with tics to hospital emergency departments during the pandemic.^7^ In the UK, information about emergency department and secondary care visits should be fed back to primary care. However, if some events were missing from the primary care record, our definition may have underestimated new tic presentations. While this may reduce the precision of our estimates, we believe systematic bias is unlikely given that the same methods were used for all study participants. Records of new-onset tic symptoms were extracted using a clinical code list which was reviewed and confirmed by one of the co-authors (CH), a consultant child and adolescent psychiatrist with expertise in tic disorders. Details of these codes are provided in the Supplementary Table S1. However, the accuracy of these codes has not been verified by independent clinical assessment. We were unable to categorise tic symptoms as arising from a tic disorder or representing functional tic-like symptoms.^1^, ^2^, ^3^^,^^12^ In addition, the study did not include data on environmental and contextual factors such as family disruption, adequacy of home-based educational provision during school closures, and pandemic-related changes in online and social media activity. The size of the sample with tic symptoms was not sufficient to allow sub-group analysis for some of the co-occurring mental health symptoms and conditions. Finally, this study is based on an observational study design and so we are unable to infer causal relationships.

This is the first study, to our knowledge, to examine the impact of the COVID-19 pandemic on the incidence of tics in children and young people in a large, representative, population-based sample. Previous studies have focussed on specific clinical populations (e.g. young people presenting to emergency departments)^7^ but these include only a sub-sample of all clinical presentations of tics. As we would expect the majority of cases to be captured in general practice records even if the initial presentation was elsewhere, our study is likely to be more representative of all young people with tic symptoms. One study^18^ that included measures of tics before and during the pandemic focussed on young people with a tic disorder diagnosis enrolled in the active control arm of a large clinical trial of online behavioural therapy for tics, which spanned the onset of the pandemic in 2020.^19^ This study found no increase in tic severity when comparing pre- and during pandemic measures, suggesting that children and young people with existing tic disorders (including Tourette syndrome) experienced, at most, a modest effect of the pandemic on their tics.

The main contribution of our large population-based cohort study is that it provides, for the first time, robust evidence of a marked increase in tic symptoms during the pandemic specifically affecting teenage girls, and an estimate of the number of young people affected. The explanation for this dramatic rise in tics in teenage girls remains unclear and will require further research. Our finding of a significant increase in rates of emotional symptoms (anxiety, depression, self-harm and eating disorders) in those young people with first-onset tics during the pandemic compared to the pre-pandemic period suggests that the increased incidence of tics in teenage girls may be related to emotional and social impacts of the pandemic experienced most acutely in teenage girls. Although we were unable to distinguish between tic symptoms arising from tic disorders and functional tics, the increase in new-onset tics in this population-based cohort mirrors the clinical descriptions in the literature of a rise in functional tic presentations in teenage girls during the pandemic.^2^^,^^4^^,^^12^ In contrast, we did not report a rise in tic intensity during the pandemic in children and young people enrolled in a clinical trial control arm with a pre-existing diagnosis of a tic disorder.^18^ Although numbers were too low to compare changes in rates of comorbidities between sub-groups, in teenage girls we found that those with new onset tics during the pandemic had higher rates of anxiety and lower rates of ADHD comorbidity compared to those presenting pre-pandemic, which would support reports of an increase in functional tics in teenage girls.^4^

Our study provided estimates of monthly incidence rates which could be related to the timing of national UK lockdowns and school closures (Fig. 1). Interestingly, a rise in tic incidence was not observed at the beginning of the pandemic in the first national lockdown. This may, in part, be explained by a lack of access to health services during this period. The peak incidence of tics in teenage girls was recorded towards the end of the third national lockdown in March 2021. Further research is required into the contextual factors, including social media activity and reduced face to face peer contact, which may have been related to increased tic presentations in vulnerable young people at this time.^8^

In conclusion, this large population-based study provides evidence to support anecdotal clinical observations that there has been a marked increase in the incidence of tics in teenage girls during the COVID-19 pandemic. This rise in sudden onset of tics in teenage girls associated with an increase in coexisting anxiety and emotional symptoms suggests that clinicians should consider functional tics as part of the differential diagnosis of new onset tics in teenage girls.^1^, ^2^, ^3^, ^4^ Further research will be required to understand the key contextual and causal processes underpinning this rise in tics in teenage girls during the pandemic and what clinical interventions and approaches are most helpful for these young people.

## Contributors

CH came up with the original research question and secured the funding. RHJ, RMJ and CACC developed the study's methodology. RHJ and RMJ were responsible for the data analysis and verification of the data. CLH, RHJ, RMJ and CH wrote the first draft of the manuscript. All authors interpreted the data, reviewed and edited the final version of the manuscript. All authors had full access to all the data in the study and accept responsibility for the decision to submit for publication.

## Data sharing statement

The data used in this study were provided under licence by CPRD (www.cprd.com) and cannot be shared by the authors. The code lists used to define tics, ethnicity, and other covariates are available via https://clinicalcodes.rss.mhs.man.ac.uk/. All code lists and the statistical code (in the form of Stata do-files) used to prepare and analyse the data are available on Zenodo.org (https://doi.org/10.5281/zenodo.6951755).

## Declaration of interests

All authors declare no competing interests.
