# Supplementary material for: Impact of the COVID-19 pandemic on incidence of tics in children and young people: a population-based cohort study
Source: eClinicalMedicine. 2023 Feb 16;57:101857. doi: 10.1016/j.eclinm.2023.101857 (PMC9932691; doi:10.1016/j.eclinm.2023.101857)
Supplement: Supplementary Tables S1–S5 [file mmc1.docx]

Supplementary Material

Table of Contents

[Supplementary Table 1, Clinical codes used to extract tic records from the CPRD Aurum dataset 1](#_Toc121315545)

[Supplementary Table 2. Conditions present at the time of first tic record, by age and sex 2](#_Toc121315546)

[Supplementary Table 3. Annual incidence rates of tics per 10,000 person-years by age and sex, 2015 to 2021 3](#_Toc121315547)

[Supplementary Table 4. Negative binomial regression comparing the incidence of tics between time periods and age-sex groups, including an interaction between time period and age-sex group 4](#_Toc121315548)

[Supplementary Table 5. Negative binomial regression comparing the incidence of tics between time periods and age-sex groups adjusting for deprivation and region, and including an interaction between time period and age-sex group 5](#_Toc121315549)

# Supplementary Table 1, Clinical codes used to extract tic records from the CPRD Aurum dataset

Proprietary EMIS codes (part of the EMIS® software system) have been masked. The code descriptions are shown exactly as they appear in the CPRD medical dictionary. CPRD Clinical Practice Research Datalink. Only the codes marked with an asterisk (*) were present in the dataset.

| **Read code** | **SNOMED CT code** | **Description** |
| --- | --- | --- |
| 1B24.00 | 1480910015 | Has a tic* |
| 1B24.11 | 1480910015 | Tic – symptom* |
| 2974.00 | 254925012 | O/E - spasm/tic* |
| 2974.12 | 254926013 | O/E - tic* |
| E272.00 | 1491776015 | Tics* |
| E272.99 |  | Psychogenic tics |
| E272000 | 2023019 | Tic disorder unspecified* |
| E272100 | 295400012 | Transient childhood tic* |
| E272200 | 13896014 | Chronic motor tic disorder* |
| E272300 | 9646017 | Gilles de la Tourette's disorder* |
| E272z00 | 1480910015 | Tic NOS* |
| Eu95.00 | 2023019 | [X]Tic disorders* |
| Eu95.99 |  | Tic disorder |
| Eu95000 | 94081014 | [X]Transient tic disorder* |
| Eu95100 | 2023019 | [X]Chronic motor or vocal tic disorder* |
| Eu95200 | 9646017 | [X]Comb vocal multiple motor tic disorder - de la Tourette* |
| Eu95y00 | 2023019 | [X]Other tic disorders* |
| Eu95z00 | 2023019 | [X]Tic disorder, unspecified* |
| F133.00 | 297069015 | Tics of organic origin* |
| F133.99 |  | Organic tic |
| [EMIS] |  | Facial tic |
| [EMIS] |  | Gilles de la Tourette's syndrome |
| [EMIS] | 194918012 | Tourette's syndrome* |
| [EMIS] | 194919016 | Tourette's disorder* |
| [EMIS] | 194920010 | Combined vocal and multiple motor tic disorder* |
| [EMIS] | 2668439013 | On examination - spasm/tic |
| [EMIS] | 2837918016 | Gilles de la Tourette syndrome |
| [EMIS] | 2921034018 | Gilles de la Tourettes syndrome |
| [EMIS] | 94081014 | Transient tic disorder* |
| [EMIS] | 964921000000113 | [X]Tic disorders |

# Supplementary Table 2. Conditions present at the time of first tic record, by age and sex

Some rows have been omitted to mask small cell counts. ADHD attention deficit hyperactivity disorder.

|  | **2015 to 2019** | | **2020** | | **2021** | |  |  |
| --- | --- | --- | --- | --- | --- | --- | --- | --- |
|  | N | (%) | N | (%) | N | (%) | Chi^2^ | p-value |
| **Male 4 to 11 years** | 4,067 | ꞏꞏ | 804 | ꞏꞏ | 899 | ꞏꞏ | ꞏꞏ | ꞏꞏ |
| ADHD | 373 | (9ꞏ2%) | 66 | (8ꞏ2%) | 91 | (10ꞏ1%) | 1ꞏ87 | 0ꞏ393 |
| Anxiety (phobic or generalised) | 128 | (3ꞏ1%) | 48 | (6ꞏ0%) | 40 | (4ꞏ4%) | 16ꞏ32 | <0ꞏ001 |
| Autism Spectrum Disorder | 319 | (7ꞏ8%) | 62 | (7ꞏ7%) | 77 | (8ꞏ6%) | 0ꞏ59 | 0ꞏ745 |
| **Male 12 to 18 years** | 1,078 | ꞏꞏ | 234 | ꞏꞏ | 311 | ꞏꞏ | ꞏꞏ | ꞏꞏ |
| ADHD | 227 | (21ꞏ1%) | 54 | (23ꞏ1%) | 60 | (19ꞏ3%) | 1ꞏ16 | 0ꞏ561 |
| Anxiety (phobic or generalised) | 132 | (12ꞏ2%) | 42 | (17ꞏ9%) | 52 | (16ꞏ7%) | 7ꞏ73 | 0ꞏ021 |
| Autism Spectrum Disorder | 191 | (17ꞏ7%) | 55 | (23ꞏ5%) | 63 | (20ꞏ3%) | 4ꞏ55 | 0ꞏ103 |
| Self-harm (intentional or unspecified) | 14 | (1ꞏ3%) | 6 | (2ꞏ6%) | 10 | (3ꞏ2%) | 5ꞏ66 | 0ꞏ059 |
| **Female 4 to 11 years** | 1,435 | ꞏꞏ | 334 | ꞏꞏ | 433 | ꞏꞏ | ꞏꞏ | ꞏꞏ |
| ADHD | 47 | (3ꞏ3%) | 12 | (3ꞏ6%) | 13 | (3ꞏ0%) | 0ꞏ21 | 0ꞏ901 |
| Anxiety (phobic or generalised) | 54 | (3ꞏ8%) | 18 | (5ꞏ4%) | 25 | (5ꞏ8%) | 4ꞏ10 | 0ꞏ129 |
| Autism Spectrum Disorder | 37 | (2ꞏ6%) | 10 | (3ꞏ0%) | 11 | (2ꞏ5%) | 0ꞏ20 | 0ꞏ904 |
| **Female 12 to 18 years** | 533 | ꞏꞏ | 489 | ꞏꞏ | 628 | ꞏꞏ | ꞏꞏ | ꞏꞏ |
| ADHD | 33 | (6ꞏ2%) | 14 | (2ꞏ9%) | 31 | (4ꞏ9%) | 6ꞏ37 | 0ꞏ041 |
| Anxiety (phobic or generalised) | 93 | (17ꞏ4%) | 118 | (24ꞏ1%) | 179 | (28ꞏ5%) | 19ꞏ61 | <0ꞏ001 |
| Autism Spectrum Disorder | 36 | (6ꞏ8%) | 31 | (6ꞏ3%) | 51 | (8ꞏ1%) | 1ꞏ50 | 0ꞏ472 |
| Depression | 24 | (4ꞏ5%) | 28 | (5ꞏ7%) | 54 | (8ꞏ6%) | 8ꞏ61 | 0ꞏ014 |
| Eating disorder | 10 | (1ꞏ9%) | 12 | (2ꞏ5%) | 23 | (3ꞏ7%) | 3ꞏ66 | 0ꞏ160 |
| Self-harm (intentional or unspecified) | 30 | (5ꞏ6%) | 41 | (8ꞏ4%) | 59 | (9ꞏ4%) | 5ꞏ88 | 0ꞏ053 |

# Supplementary Table 3. Annual incidence rates of tics per 10,000 person-years by age and sex, 2015 to 2021

|  | **Incidence rate (95% confidence interval) per 10,000 person-years** | | | |
| --- | --- | --- | --- | --- |
| **Year** | **Males 4 to 11 years** | **Males 12 to 18 years** | **Females 4 to 11 years** | **Females 12 to 18 years** |
| 2015 | 12ꞏ23 (11ꞏ37 to 13ꞏ16) | 3ꞏ86 (3ꞏ32 to 4ꞏ49) | 4ꞏ75 (4ꞏ21 to 5ꞏ36) | 2ꞏ27 (1ꞏ84 to 2ꞏ80) |
| 2016 | 13ꞏ13 (12ꞏ25 to 14ꞏ08) | 4ꞏ11 (3ꞏ56 to 4ꞏ74) | 5ꞏ02 (4ꞏ47 to 5ꞏ63) | 1ꞏ68 (1ꞏ32 to 2ꞏ13) |
| 2017 | 13ꞏ58 (12ꞏ69 to 14ꞏ54) | 4ꞏ61 (4ꞏ03 to 5ꞏ27) | 4ꞏ97 (4ꞏ43 to 5ꞏ57) | 2ꞏ15 (1ꞏ76 to 2ꞏ64) |
| 2018 | 13ꞏ92 (13ꞏ03 to 14ꞏ88) | 5ꞏ32 (4ꞏ71 to 6ꞏ01) | 5ꞏ21 (4ꞏ67 to 5ꞏ83) | 2ꞏ85 (2ꞏ40 to 3ꞏ39) |
| 2019 | 13ꞏ87 (12ꞏ98 to 14ꞏ83) | 5ꞏ07 (4ꞏ48 to 5ꞏ74) | 4ꞏ70 (4ꞏ18 to 5ꞏ29) | 3ꞏ41 (2ꞏ92 to 3ꞏ99) |
| 2020 | 13ꞏ19 (12ꞏ31 to 14ꞏ14) | 4ꞏ68 (4ꞏ12 to 5ꞏ32) | 5ꞏ72 (5ꞏ14 to 6ꞏ37) | 10ꞏ32 (9ꞏ45 to 11ꞏ28) |
| 2021 | 15ꞏ09 (14ꞏ13 to 16ꞏ11) | 6ꞏ16 (5ꞏ51 to 6ꞏ88) | 7ꞏ59 (6ꞏ91 to 8ꞏ34) | 13ꞏ07 (12ꞏ09 to 14ꞏ14) |

# Supplementary Table 4. Negative binomial regression comparing the incidence of tics between time periods and age-sex groups, including an interaction between time period and age-sex group

IRR Incidence rate ratio, CI confidence interval

|  | **IRR** | **95% CI** | **p-value** |
| --- | --- | --- | --- |
| **Age-sex group** |  |  |  |
| Males 4 to 11 years | 1ꞏ00 | ꞏꞏ | ꞏꞏ |
| Males 12 to 18 years | 0ꞏ34 | (0ꞏ32 to 0ꞏ37) | <0ꞏ0001 |
| Females 4 to 11 years | 0ꞏ36 | (0ꞏ34 to 0ꞏ39) | <0ꞏ0001 |
| Females 12 to 18 years | 0ꞏ18 | (0ꞏ17 to 0ꞏ20) | <0ꞏ0001 |
| **Time period** |  |  |  |
| 2015-2019 | 1ꞏ00 | ꞏꞏ | ꞏꞏ |
| 2020 | 0ꞏ99 | (0ꞏ91 to 1ꞏ09) | 0ꞏ9137 |
| 2021 | 1ꞏ14 | (1ꞏ04 to 1ꞏ24) | 0ꞏ0043 |
| **Age-sex group x time period** |  |  |  |
| Males 4 to 11 years x 2015-2019 | 1ꞏ00 | ꞏꞏ | ꞏꞏ |
| Males 4 to 11 years x 2020 | 1ꞏ00 | ꞏꞏ | ꞏꞏ |
| Males 4 to 11 years x 2021 | 1ꞏ00 | ꞏꞏ | ꞏꞏ |
| Males 12 to 18 years x 2015-2019 | 1ꞏ00 | ꞏꞏ | ꞏꞏ |
| Males 12 to 18 years x 2020 | 1ꞏ03 | (0ꞏ87 to 1ꞏ23) | 0ꞏ7191 |
| Males 12 to 18 years x 2021 | 1ꞏ19 | (1ꞏ01 to 1ꞏ40) | 0ꞏ0388 |
| Females 4 to 11 years x 2015-2019 | 1ꞏ00 | ꞏꞏ | ꞏꞏ |
| Females 4 to 11 years x 2020 | 1ꞏ18 | (1ꞏ01 to 1ꞏ39) | 0ꞏ0375 |
| Females 4 to 11 years x 2021 | 1ꞏ37 | (1ꞏ18 to 1ꞏ58) | <0ꞏ0001 |
| Females 12 to 18 years x 2015-2019 | 1ꞏ00 | ꞏꞏ | ꞏꞏ |
| Females 12 to 18 years x 2020 | 4ꞏ18 | (3ꞏ55 to 4ꞏ92) | <0ꞏ0001 |
| Females 12 to 18 years x 2021 | 4ꞏ67 | (4ꞏ00 to 5ꞏ46) | <0ꞏ0001 |

# Supplementary Table 5. Negative binomial regression comparing the incidence of tics between time periods and age-sex groups adjusting for deprivation and region, and including an interaction between time period and age-sex group

|  | **IRR** | **95% CI** | **p-value** |
| --- | --- | --- | --- |
| **Age-sex group** |  |  |  |
| Males 4 to 11 years | 1ꞏ00 | ꞏꞏ | ꞏꞏ |
| Males 12 to 18 years | 0ꞏ34 | (0ꞏ32 to 0ꞏ37) | <0ꞏ0001 |
| Females 4 to 11 years | 0ꞏ37 | (0ꞏ34 to 0ꞏ39) | <0ꞏ0001 |
| Females 12 to 18 years | 0ꞏ19 | (0ꞏ17 to 0ꞏ20) | <0ꞏ0001 |
| **Time period** |  |  |  |
| 2015-2019 | 1ꞏ00 | ꞏꞏ | ꞏꞏ |
| 2020 | 0ꞏ99 | (0ꞏ91 to 1ꞏ08) | 0ꞏ8972 |
| 2021 | 1ꞏ14 | (1ꞏ05 to 1ꞏ23) | 0ꞏ0018 |
| **Age-sex group x time period** |  |  |  |
| Males 4 to 11 years x 2015-2019 | 1ꞏ00 | ꞏꞏ | ꞏꞏ |
| Males 4 to 11 years x 2020 | 1ꞏ00 | ꞏꞏ | ꞏꞏ |
| Males 4 to 11 years x 2021 | 1ꞏ00 | ꞏꞏ | ꞏꞏ |
| Males 12 to 18 years x 2015-2019 | 1ꞏ00 | ꞏꞏ | ꞏꞏ |
| Males 12 to 18 years x 2020 | 1ꞏ03 | (0ꞏ87 to 1ꞏ22) | 0ꞏ7247 |
| Males 12 to 18 years x 2021 | 1ꞏ19 | (1ꞏ02 to 1ꞏ39) | 0ꞏ0293 |
| Females 4 to 11 years x 2015-2019 | 1ꞏ00 | ꞏꞏ | ꞏꞏ |
| Females 4 to 11 years x 2020 | 1ꞏ19 | (1ꞏ02 to 1ꞏ38) | 0ꞏ0270 |
| Females 4 to 11 years x 2021 | 1ꞏ37 | (1ꞏ19 to 1ꞏ58) | <0ꞏ0001 |
| Females 12 to 18 years x 2015-2019 | 1ꞏ00 | ꞏꞏ | ꞏꞏ |
| Females 12 to 18 years x 2020 | 4ꞏ16 | (3ꞏ57 to 4ꞏ86) | <0ꞏ0001 |
| Females 12 to 18 years x 2021 | 4ꞏ65 | (4ꞏ02 to 5ꞏ39) | <0ꞏ0001 |
| **Deprivation quintile (IMD)** |  |  |  |
| 1 (least deprived) | 1ꞏ00 |  |  |
| 2 | 1ꞏ00 | (0ꞏ94 to 1ꞏ07) | 0ꞏ8840 |
| 3 | 0ꞏ95 | (0ꞏ89 to 1ꞏ01) | 0ꞏ1089 |
| 4 | 0ꞏ85 | (0ꞏ79 to 0ꞏ90) | <0ꞏ0001 |
| 5 (most deprived) | 0ꞏ77 | (0ꞏ72 to 0ꞏ82) | <0ꞏ0001 |
| Not known | 0ꞏ96 | (0ꞏ81 to 1ꞏ13) | 0ꞏ5847 |
| **Practice region** |  |  |  |
| North East | 1ꞏ00 |  |  |
| North West | 0ꞏ77 | (0ꞏ68 to 0ꞏ86) | <0ꞏ0001 |
| Yorkshire and The Humber | 0ꞏ85 | (0ꞏ74 to 0ꞏ98) | 0ꞏ0286 |
| East Midlands | 0ꞏ92 | (0ꞏ78 to 1ꞏ08) | 0ꞏ3020 |
| West Midlands | 0ꞏ71 | (0ꞏ64 to 0ꞏ80) | <0ꞏ0001 |
| East of England | 0ꞏ91 | (0ꞏ80 to 1ꞏ04) | 0ꞏ1487 |
| London | 0ꞏ52 | (0ꞏ47 to 0ꞏ59) | <0ꞏ0001 |
| South East | 0ꞏ92 | (0ꞏ82 to 1ꞏ03) | 0ꞏ1333 |
| South West | 1ꞏ07 | (0ꞏ96 to 1ꞏ20) | 0ꞏ2224 |
